# PDE4 and Epac1 Synergistically Promote Rectal Carcinoma via the cAMP Pathway

**DOI:** 10.1155/2019/7145198

**Published:** 2019-01-23

**Authors:** Xiangyu Kong, Ganghao Ai, Dai Wang, Renzhen Chen, Dongbei Guo, Youliang Yao, Kai Wang, Guiye Liang, Fengjie Qi, Wenzhi Liu, Yongxing Zhang

**Affiliations:** ^1^Department of Gastrointestinal Surgery, Affiliated Zhongshan Hospital of Dalian University, Dalian, 116001 Liaoning, China; ^2^State Key Laboratory of Molecular Vaccinology and Molecular Diagnostics, School of Public Health, Xiamen University, Xiamen, 361102 Fujian, China; ^3^Department of Pathology, The third Affiliated Hospital of Shenzhen University, Shenzhen, Guangdong 518001, China

## Abstract

**Objective:**

To assess the expression levels of exchange protein 1 directly activated by cAMP (Epac1) and phosphodiesterase 4 (PDE4) in rectal carcinoma, and their associations with clinicopathological indexes. In addition, the associations of PDE4 and Epac1 with A-kinase anchor protein 95, connexin 43, cyclin D1, and cyclin E1 were evaluated.

**Methods:**

The PV-9000 two-step immunohistochemistry method was used to determine protein expression in 44 rectal carcinoma tissue samples and 16 paracarcinoma tissue specimens.

**Results:**

The positive rate of PDE4 protein expression in rectal carcinoma tissues was higher than that of paracarcinoma tissues (59.09% vs. 12.5%, *P* < 0.05). Similar findings were obtained for Epac1 (55% vs. 6.25%, *P* < 0.05). No significant associations of PDE4 and Epac1 with degree of differentiation, histological type, and lymph node metastasis were found in rectal carcinoma (*P* > 0.05). Correlations between PDE4 and Epac1, PDE4 and Cx43, PDE4 and cyclin E1, and Epac1 and Cx43 were observed (all *P* < 0.05). There was no correlation between the other protein pairs examined (*P* > 0.05).

**Conclusion:**

PDE4 and Epac1 expression levels are increased in rectal carcinoma tissues, suggesting that the two proteins may be involved in the development of this malignancy. Meanwhile, correlations between PDE4 and Epac1, PDE4 and Cx43, PDE4 and cyclin E1, and Epac1 and Cx43 suggested synergistic effects of these proteins in promoting rectal carcinoma.

## 1. Introduction

Signal transduction is a necessary process for cells to achieve normal physiological processes. The PDE4 enzyme specifically hydrolyzes cAMP and reduces cAMP levels in the cell, to allow cAMP-dependent proteins to modulate cell signal transduction [1]. PKA, which is a downstream of cAMP signal pathway, is an important protein kinase. AKAP95 is a PKA-anchored protein that anchors PKA RII subunits; the anchored PKA can catalytically target protein phosphorylation, ensuring and expanding signal transduction by the cAMP pathway [2, 3]. Cyclin D and cyclin E proteins can promote cell proliferation at the G1 phase in mammalians, while AKAP95 as an intermediary can help cyclin D/E and PKA RII subunits from the cyclin D/E-AKAP95-PKA complex [4]. The novel exchange protein directly activated by cAMP (Epac) is a multifunctional molecule that participates in a variety of cellular processes [5]. The Epac protein includes two subtypes, i.e., Epac1 and Epac2, both of which are expressed in many tissues and organs [6, 7]. Different organs and distinct developmental stages also show differences [8]. We have previously reported the combinatory relationship between AKAP95 and Cx43 in the cell cycle [9]. Studies mentioned that Epac can regulate Cx43 to promote gap junction formation and intercellular communication [10].

The above findings suggest that PDE4, Epac, AKAP95, Cx43, and cyclin D/E have some associations. The immunohistochemical method was used to assess the protein expression of PDE4 and Epac1 in 44 samples of rectal carcinoma alongside 16 paracarcinoma tissue samples. The associations of various proteins were analyzed.

## 2. Materials and Methods

### 2.1. Tumor Sources

Tissue samples from 44 cases with definite pathological diagnosis of invasive rectal carcinoma were collected from the First Affiliated Hospital of Liaoning Medical University. Patient age ranged between 39 and 79 years, averaging 60 ± 8; there were 28 males and 16 females. A total of 38, 4, and 2 patients had tubular or papillary adenocarcinoma, mucinous adenocarcinoma, and signet-ring cell carcinoma, respectively. Cancer cells were highly, moderately, and poorly differentiated in 4, 36, and 4 patients, respectively. A total of 23 patients had lymph node metastasis; 15 presented no lymph node metastasis, while the lymph node metastasis status was unclear for the remaining 6 individuals. In addition, paracarcinoma tissues were obtained from normal rectal tissues at least 3 cm away from cancerous tissues, in 16 of the 44 patients. Pathological examination was also performed on the paracarcinoma tissues to confirm the absence of cancer cells. The study protocol was approved by the Medical Ethics Committee of the School of Public Health in Xiamen University, China.

### 2.2. Reagents and Methods

All specimens were fixed in 10% neutral formaldehyde, paraffin embedded, and sliced into continuous sections of 4 *μ*m. The PV-9000 two-step immunohistochemical staining kit (Zhongshan Jinqiao Biotechnology Company, Beijing, China) was used for protein expression analysis, according to the manufacturer's instructions. The assay involved DAB staining and hematoxylin counterstaining. Rabbit anti-human Epac1 antibodies were purchased from Abcam (Cambridge, UK), whereas mouse anti-human PDE4 monoclonal antibodies were from Santa Cruz (Dallas, Texas, USA). PBS was used as the negative control sample.

### 2.3. Criteria for Judging Positive Expression

A brown–yellow stain was considered positive expression of the protein, while the absence of brown–yellow staining indicated no protein expression. Ten different high-power fields were randomly evaluated per section, with 200 tumor cells counted per field. The ratio of positive to total cells was used as a metric to assess positive protein expression: “−,” <10% brown; “±,” ≥10% and <25%; “+,” ≥25% and <50%; “++,” ≥50% and <75%; and “+++,” ≥75%. For data analysis, “−” and “±” were considered negative expression and “+,” “++,” and “+++” indicated positive expression.

## 3. Statistical Analysis

Positive rates of protein expression were assessed by the *χ*^2^ test and were assessed by Spearman's rank correlation analysis. *P* < 0.05 was considered statistically significant, and data were analyzed with the SPSS13.0 software.

## 4. Results

### 4.1. PDE4 and Epac1 Protein Levels in Rectal Carcinoma Tissues

We previously assessed rectal carcinoma tissues and found higher positive rates of AKAP95, cyclin E1, and cyclin D1 compared with paracarcinoma tissues. Meanwhile, the positive rate of Cx43 was lower than that of paracarcinoma tissues. These findings suggested that AKAP95, cyclin D1, and cyclin E1 may promote cancer, while the Cx43 protein suppresses cancer development [11].

**Figure 1:**
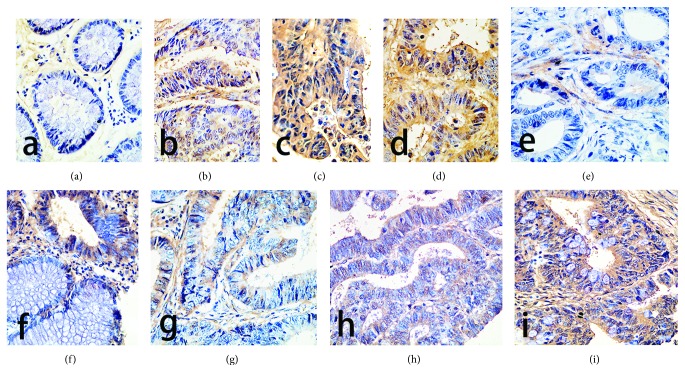
Epac1 and PDE4 expression in rectal carcinoma tissues (×400). The subfigures (a), (b), (c), and (d) indicated the protein expression of Epac1 in rectal cancer tissues. (a) No expression. (b) Moderately expression. (c, d) High expression levels, expression in the cytoplasm and in the nucleus. The subfigures (e), (f), (g), (h), and (i) indicated the protein expression of PDE4 in rectal cancer tissues. (e) No expression. (f) The top of the picture is moderately expression, and the bottom is no expression. (g) Low expression levels. (h) Moderately expression. (i) High expression levels, mainly in the cytoplasm, with low amounts in the nucleus.

In this study, PDE4 and Epac1 protein levels were further assessed in rectal cancer samples. PDE4 and Epac1 protein levels in 44 rectal cancer and 16 paracarcinoma tissue samples were assessed (Table 1). The positive expression rate for PDE4 in rectal cancer was 59.09% (26/44), which was higher than that of paracarcinoma tissue specimens (12.5%, 2/16, *P* < 0.05). The positive expression rate for Epac1 in rectal cancer was 55.00% (24/44), which was also higher than that of paracarcinoma tissue samples (6.25%, 1/16, *P* < 0.05). The PDE4 and Epac1 proteins were expressed in the cytoplasm of rectal carcinoma tissues and with expression obtained in nuclei (Figure 1).

There were no associations of PDE4 and Epac1 with degree of differentiation, histological type, and lymph node metastasis in rectal carcinoma (*P* > 0.05).

### 4.2. Associations of PDE4, Epac1, AKAP95, Cx43, Cyclin E1, and Cyclin D1 in Rectal Carcinoma Tissues

The associations of PDE4 and Epac1 with AKAP95, Cx43, cyclin E1, and cyclin D1 in 44 rectal carcinoma samples were assessed. The results indicated correlations between PDE4 and Epac1 (Table 2), PDE4 and cyclin E1 (Table 3), PDE4 and Cx43 (Table 4), and Epac1 and Cx43 (Table 5) (*P* < 0.05). No significant associations were obtained for the remaining protein pairs (data not shown).

## 5. Discussion

The Epac protein is mainly involved in cell adhesion, communication, secretion, and differentiation [5]. As a multifunctional signaling molecule, it has multiple unique regulatory functions, in the immune, respiratory, nervous, cardiovascular, and endocrine systems [12, 13]. Epac1 exists in all organisms, and the Epac2 protein is found only in gland tissues and the nervous system [14]. It was recently reported that the Epac inhibitor ESI-09 suppresses the proliferation of pancreatic tumor cells [15]; meanwhile, Epac1 is highly expressed in gastric cancer cells [16]. This study also demonstrated high Epac1 expression levels in rectal cancer tissue samples, which may indicate that this protein promotes cancer in the digestive system.

PDE is an enzyme involved in cAMP hydrolysis. PDE4 is a member of the PDE family of proteins; it specifically hydrolyzes cAMP, to enable inactivation by 5′-AMP formation, which ends the downstream signal transduction [17]. PDE4 is associated with the occurrence of multiple tumors and highly expressed in a variety of tumor tissues [18]. PDE4 showed high expression levels in rectal cancer tissues, which may suggest that it promotes rectal cancer as well, corroborating previous reports.

Meanwhile, PDE4 can bind and activate the downstream Epac protein, which is the downstream target of cAMP. Epac binds to cAMP with high affinity and activates the Ras superfamily small GTPases Rap1 and Rap2, indicating the existence of the PDE4/cAMP/Epac1 signaling pathway [18]. As shown above, correlations were found between PDE4 and Epac1, PDE4 and Cx43, and Epac1 and Cx43 (*P* < 0.05). These findings indicate possible interactions of the PDE4, Epac1, and Cx43 proteins, which commonly play roles in rectal cancer occurrence.

This study also demonstrated a correlation between PDE4 and cyclin E1. Cyclin E1 is an important protein in the G1/S stage of the cell cycle. The association of PDE4 with cyclin E1 suggests that the two proteins may exert synergistic effects on the G1/S transition during cell proliferation. However, there was no correlation between PDE4 and cyclin D1.

The Epac protein of rabbit myocardial cells regulates Cx43 phosphorylation at ser368, which affects Cx43 protein function [10]. Studies confirmed that Cx43 plays a role of tumor inhibition in multiple organisms [19–22]. In this study, Epac1 level was associated with Cx43 expression, further illustrating a close relationship for inducing rectal cancer.

In T cell leukemia patients, PDE4 and AKAP95 levels are associated in T cells [23]. However, as shown above, no correlation between PDE4 and AKAP95 was found in rectal cancer tissues. The discrepancy caused by the histological differences remains unclear. In rat myocardial cells, mAKAP, which is a member of the AKAP family, can bind with Epac1 to regulate signaling and modulate rat myocardial hypertrophy [24]. Our experimental study revealed no correlation between Epac1 and AKAP95 protein levels. This phenomenon may be associated with tumorigenesis, related to different members of the AKAP family or histological differences. The specific mechanism requires further assessment.

In summary, there are some close correlations in PDE4, Epac1, cyclin E1, and Cx43 in rectal cancer. This may be an important network of proteins for regulating cell cycle in rectal cancer. And it may become a new therapeutic target in the future. However, this needs further study.

## Figures and Tables

**Table 1:** PDE4 and Epac1 protein levels in rectal cancer tissues.

Protein	Features	Rectal cancer	Paracarcinoma tissues	*χ* ^2^	*P*
Epac1	Positive	24	1	11.260	0.01
	Negative	20	15		
PDE4	Positive	26	2	10.233	0.01
	Negative	18	14		

Note: in rectal cancer and paracarcinoma tissues, PDE4 and Epac1 levels showed a statistically significant difference.

**Table 2:** Correlation between PDE4 and Epac1 protein levels in rectal cancer.

Epac1	PDE4					*r*_*s*_	*P*
	−	+−	+	++	+++		
−	5	1	1	1	0	0.419	0.005
+−	3	4	5	0	0		
+	2	0	5	3	1		
++	0	1	3	5	0		
+++	1	1	2	0	0		

Note: *r*_*s*_ is the spearman rank correlation coefficient.

**Table 3:** Correlation between PDE4 and cyclin E1 protein levels in rectal cancer.

Cyclin E1	PDE4					*r*_*s*_	*P*
	−	+−	+	++	+++		
−	5	1	3	1	0	0.300	0.048
+−	0	2	3	3	0		
+	5	2	3	0	0		
++	1	0	6	2	1		
+++	0	2	1	3	0		

Note: *r*_*s*_ is the spearman rank correlation coefficient.

**Table 4:** Correlation between PDE4 and Cx43 protein levels in rectal cancer.

PDE4	Cx43					*r*_*s*_	*P*
	−	+−	+	++	+++		
−	6	3	2	0	0	0.367	0.014
+−	1	5	1	0	0		
+	2	7	7	0	0		
++	2	2	2	2	1		
+++	0	1	0	0	0		

Note: *r*_*s*_ is the spearman rank correlation coefficient.

**Table 5:** Correlation between Epac1 and Cx43 protein levels in rectal cancer.

Epac1	Cx43					*r_s_*	*P*
	−	+−	+	++	+++		
−	5	2	1	0	0	0.360	0.016
+−	4	3	5	0	0		
+	1	7	2	1	0		
++	1	3	3	1	1		
+++	0	3	1	0	0		

Note: *r*_*s*_ is the spearman rank correlation coefficient.

## Data Availability

(1) The data of AKAP95, Cx43, cyclin E1, and cyclin D1 expression in rectal carcinoma tissues used to support the findings of this study have been deposited in the PubMed repository [PMID: 25973052 or PMCID: PMC4396224]. The prior studies are cited at relevant places within the text as references [11]. (2) The data of associations of PDE4 and Epac1 with AKAP95, Cx43, cyclin E1, and cyclin D1 used to support the findings of this study are included within the article.
